# Case report: ‘Atypical Richter transformation from CLL-type monoclonal B-cell lymphocytosis into Burkitt lymphoma in a treatment naïve patient’

**DOI:** 10.3389/fonc.2024.1296238

**Published:** 2024-05-03

**Authors:** Annaïse J. Jauch, Ilaria Alborelli, Andreas Reusser, Albert Baschong, Cyrill Rütsche, Olivier Bignucolo, Jakob Passweg, Stefan Dirnhofer, Fatime Krasniqi

**Affiliations:** ^1^ Division of Medical Oncology, University Hospital of Basel, Basel, Switzerland; ^2^ Institute of Medical Genetics & Pathology, Pathology, University Hospital Basel, Basel, Switzerland; ^3^ Division of Medical Oncology, Kantonsspital Basel-Land, Liestal, Switzerland; ^4^ Institute for Pathology, Kantonsspital Basel-Land, Liestal, Switzerland; ^5^ Division of Hematology, University Hospital Basel, Basel, Switzerland; ^6^ Swiss Institute of Bioinformatics (SIB), Department of Biomedical Sciences, University of Lausanne, Basel, Switzerland

**Keywords:** Richter transformation, MBL, SLL/CLL, Burkitt lymphoma, state sequencing

## Abstract

**Background:**

Richter transformation refers to the progression of an initially slow-growing small lymphocytic lymphoma/chronic lymphocytic leukemia (SLL/CLL) into an aggressive lymphoma, typically diffuse large B-cell lymphoma (DLBCL) or Hodgkin lymphoma.

**Case presentation:**

The patient presented with a rapid onset of localized cervical swelling, accompanied by monoclonal B-cell lymphocytosis displaying a CLL immunophenotype. The histopathological analysis identified a Burkitt lymphoma (BL) located in the submandibular gland and adjacent lymph node. The patient’s bone marrow displayed a minor infiltration of monoclonal B-cells with a CLL immunophenotype (< 10%). Molecular analysis demonstrated the presence of the same monoclonal rearrangement in the framework region (FR3 region) of the immunoglobulin heavy chain (*IGH*) locus. High-throughput sequencing of the immunoglobulin heavy and light chains also confirmed the presence of the same rearrangement in SLL/CLL and in the Burkitt lymphoma sample, but also highlighted the presence of a second rearrangement in the Burkitt lymphoma cells, not shared with the SLL/CLL cells in the bone marrow. The patient was treated with DA-EPOCH-R, which lead to a complete metabolic response.

**Conclusion:**

This report provides an exceptionally rare description of a CLL-type monoclonal B-cell lymphocytosis transforming into a very aggressive Burkitt lymphoma in a treatment naïve patient.

## Highlights

- Richter transformation refers to the progression of an initially slow-growing small lymphocytic lymphoma/chronic lymphocytic leukemia (SLL/CLL) into an aggressive lymphoma, typically diffuse large B-cell lymphoma or Hodgkin lymphoma.
- This report provides one of the first descriptions of a CLL-type monoclonal B-cell lymphocytosis transforming into a very aggressive Burkitt lymphoma, supported by high-throughput sequencing of the B-cell receptor, which demonstrated the presence of the same monoclonal rearrangement in the immunoglobulin heavy chain locus in the bone marrow and submandibular gland. Additionally, the Burkitt lymphoma cells displayed a second co-dominant rearrangement in the *IGH*.
- The patient was treated with systemic chemotherapy and both malignancies were not detectable after the treatment.

## Introduction

Richter transformation typically refers to the occurrence of an aggressive lymphoma as a secondary development in individuals with an indolent small lymphocytic lymphoma/chronic lymphocytic leukemia (SLL/CLL) (1). The annual incidence of Richter transformation is estimated to be 0.5 – 1% among SLL/CLL patients (2). The initial observation dates back to nearly a century ago when Maurice Richter observed the post-mortem findings in a man with known CLL, who had recently developed a non-tender neck swelling and fast deteriorating clinical condition. Upon microscopic lymph node examination, he documented the co-occurrence of “leukemic cells” as well as “tumor cells”. This discovery underscored the simultaneous presence of the same disease but in distinct developmental stages (3).

In 1964, Lortholary and colleagues implemented the term “Richter syndrome” to describe a phenomenon they observed in a cohort of CLL patients. These individuals experienced a declining condition characterized by the development of B symptoms, lymphocytosis and lymphadenopathy. The authors noted the co-occurrence of small lymphoid cells and large lymphoma cells (4). While histological transformation can occur in other low-grade B-cell malignancies, the term “Richter syndrome” strictly refers to this phenomenon in the context of SLL/CLL (5). The majority of SLL/CLLs transform into diffuse large B-cell lymphoma (DLBCL) (90-95%), Hodgkin lymphoma (5-10%) (1) and rarely plasmablastic lymphoma (6). *TP53* disruption and alterations in *c-MYC* are molecularly associated with Richter transformations in SLL/CLL (7). Transformation into Burkitt lymphoma (BL) has not been observed in treatment naïve SLL/CLL patients. However, two case reports have described transformations into Burkitt-like lymphomas (8, 9) and one case report described a male with CLL, which received two lines of chemo- and immunotherapy before the development of a Burkitt lymphoma (10).

The suspicion of SLL/CLL transformation to an aggressive lymphoma arises when a patient with SLL/CLL presents with newly onset of B symptoms along with rapidly growing masses, as determined through clinical examination and/or imaging studies (5). Supporting laboratory findings for Richter transformation include elevated β2 microglobulin (>2mg/L) and LDH levels (>1.5x upper reference limit). Increasing absolute lymphocytosis (≥5 x10^9^/L), thrombocytopenia (<100 x10^9^/L) and hypercalcemia (as seen in CLL patients) (7, 11–14) may as well be indicative of transformation.

Burkitt lymphoma is a highly aggressive mature B-cell lymphoma that affects both adults and children (15). Although histopathological findings support the diagnosis, genomic aberration testing is necessary for differentiating Burkitt lymphoma from other high-grade B-cell malignancies. The hallmark translocation of t(8;14)(q24;q32) rearrangement, juxtaposing the *MYC* gene to the *IGH* locus, is observed in 95% of case (15).

## Case description

**Figure 1:**
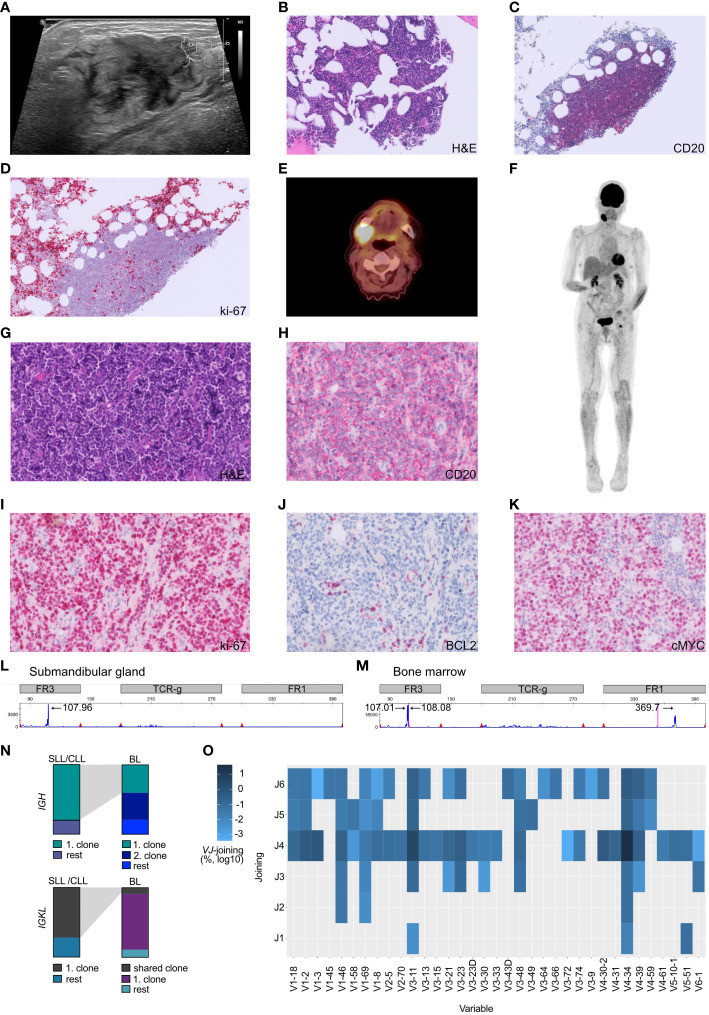
Atypical Richter transformation with localized presentation of Burkitt lymphoma in the submandibular gland. **(A)** Sonography of the cervical swelling, demonstrating inhomogeneous tissue. **(B)** The bone marrow displayed scarce nodular infiltrates of small lymphocytic cells which **(C)** stained positive for CD20^+^ and **(D)** presented a low proliferative activity (ki-67/MIB). **(E)** PET-CT scan axial plane of the patient, documented cervical consolidations with high metabolic activity. **(F)** Full body PET-CT scan with coronal plane visualizing the localized manifestation. **(G)** Submandibular gland specimen, stained with hematoxylin and eosin (H&E), demonstrating stroma with diffuse atypical lymphoid infiltrate composed of slightly pleomorphic configured cells with mitotic figures and interspersed scattered tingible body macrophages. **(H)** Submandibular gland lymphoid cells staining positive for the B-cell marker CD20. **(I)** Highly proliferative lymphoid cells (stained against ki-67) in the submandibular gland. **(J)** Submandibular gland B-cells being BCL2 negative and **(K)** c-MYC positive**. (L, M)** Multiplex PCR and high-resolution fragment analysis of the *IGH* locus. The numbers above designate fragment alignment by size distribution. A clear dominant peak was observed in the FR3 region of the *IGH* locus. This peak likely corresponds to the same clone, and is not visible in the submandibular gland sample due to higher DNA fragmentation caused by formalin-fixation (FR1 dominant peak fragment size > 360 bp). **(N)** BCR sequencing experiment. Top row) Depiction of the top *IGH* clones utilized by SLL/CLL cells *vs*. BL cells. Lower row) Depiction of the top *IGKL* clones utilized by SLL/CLL cells *vs.* BL cells. Gray shading depicts corresponding clones found in both tumors. **(O)** Heatmap illustrating the *VJ* gene usage in BL sequences not shared with the SLL/CLL B-cells and the overutilization of the *V*-segment *V4-34*. Frequency have been log10 transformed to reduce skewness of a measurement variable. Histology specimens are displayed with a 40x magnification **(A-H)** and 20x **(I-K)**.

We present a case involving an 81-year-old female, who was hospitalized in September 2022 due to the gradual development of a non-tender cervical swelling on her right side over the preceding two months. There were no accompanying symptoms of weight loss, fever, or night sweats. The patient had stopped smoking tobacco while occasionally consuming alcohol. There was no evidence of an autoimmune condition or a viral infection. Notable comorbidities were hypertensive cardiopathy with moderately impaired ejection fraction and coronary arteriosclerosis. During the physical examination, a discernible, firm, and mobile tumefaction was observed in the right cervical region. The remaining physical examination was unremarkable. Sonography revealed an inhomogeneous tissue at the site of the swelling (**Figure 1A**). In the peripheral blood a significant increase of lymphocytes 5.69 x10^9^/L was noted, while lactate dehydrogenase (LDH) levels were within the reference range (**Table 1**). Flow cytometric peripheral blood immunophenotyping showed the expansion of a mature clonal B-cell population, with restriction of the membrane immunoglobulin light-chain kappa (76% of total B-cells, absolute count 0.8 x10^9^/L). Immunoglobulin subtypes were within the reference range and no abnormal paraprotein was found *via* serum protein electrophoresis (**Table 1**). In line with the result from flow cytometric immunophenotyping, an augmentation of the free immunoglobulin light-chain kappa was measured in the serum (**Table 1**). A fine needle puncture of the swelling visualized a mixed lymphocytic infiltrate (data not shown).

**Table 1:** Hematological and immunological parameters in the peripheral blood, characterization of both tumors.

			Patient
Analysis	Unit	Reference	Female 81y
Hemoglobin	g/L	f 120-160	127
Thrombocytes	x10^9^/L	150-450	237
WBC	Leukocytes	4.5-10 x10^9^/L	11.3
	Neutrophils	1.4 - 8 x10^9^/L	4.34
	Monocytes	0.12- 0.95 x10^9^/L	0.75
	Eosinophils	< 0.7 x10^9^/L	0.19
	Basophils	< 0.2 x10^9^/L	0.05
	Total lymphocytes	1.5 - 4 x10^9^/L (20-45%)	5.69 (44.8%) ↑
% of lymphocytes	T-cells, CD3^+^	55-86%	53% ↓
	CD4^+^	33-58%	38%
	CD8^+^	13-39%	15%
	Ratio CD4^+^/CD8^+^	1-3.8	2.53
	B-cells, CD19^+^	5-22%	19%
	CD19^+^ number	0.08-0.61x10^9^/L	1.08 ↑
	NK-cells CD56^+^CD16^+^CD3^-^	5-26%	23%
Immunoglobulins			
IgG	g/L	6.64 – 14.94	7.51
IgM	g/L	0.4 – 2.3	0.91
IgA	g/L	0.7 – 4	1.64
Immunoglobulin light-chains			
Kappa	mg/L	3.3 – 19.4	24.8 ↑
Lambda	mg/L	5.7 – 26.3	15.9
K/L ratio		0.26 – 1.65	1.56
Tumor characterization		CLL/SLL in BM	Burkitt lymphoma
EBV (LMP1)		Negative	Negative
*EBV* (EBER1)		Negative	Negative
*c-MYC* translocation		Negative	Positive
Cyclin D1		NA	Positive
BCL-2		Positive	Negative
BCL-6		Negative	Positive
*BCL-2 & BCL-6* translocations		NA	Negative

Ig immunoglobulin, K/L ratio kappa/lambda ratio, NA not available, NK Natural killer, WBC white blood count. Phenotyping according to Carsetti et al. and Strati et al. (16, 17).

We conducted an ^18^F-fluorodeoxy-glucose (FDG)–positron-emission tomography and computer tomography (PET-CT) scan to investigate the possibility of additional pathological lesions in the patient. The scan visualized hypermetabolism in the right submandibular gland and an adjacent lymph node (2.6 x 3.3cm, SUV_max_ 14.9, **Figures 1B, C**.

To gain insight into the cellular origin of the rapidly growing hypermetabolic lesions, an extirpation of the cervical lymph node and a fine-needle biopsy of the submandibular gland was performed two days after the PET-CT scan. The biopsy revealed an infiltration by medium-sized, monomorphic, and blastic CD20^+^ B-cells with basophilic cytoplasm accompanied by a significant number of mitotic figures (**Figures 1D, E**). The proliferation rate was very high (ki-67/MIB 95%, **Figure 1F**) and tingible body macrophages were interspersed (**Figure 1G**). The B-cells displayed an immunophenotype characteristic of mature germinal-center B-cells, including high membrane IgM expression with kappa light-chain restriction. Additionally, the cells stained positive for CD79A, CD10, CD38, BCL6 and weakly positive for PAX5. BCL2, LMO2 and TdT were negative within B-cells, whereas c-MYC positivity was abundant (**Figure 1H**). B-cell infection by EBV was negative (**Table 1**). The pattern pointed to the differential diagnosis of Burkitt lymphoma (15).

In October 2022, a bone marrow investigation revealed only 10% infiltration by CD20^+^ small lymphoid cells (**Figures 1I, J**). These small B-cells were low proliferative (**Figure 1K**). The flow cytometric characterization showed a CLL immunophenotype (CD19^+^ CD20^dim^ CD5^+^ CD23^+^ CD200^+^ CD79b^-^ FMC7^-^ IgM^dim^), thus a Matutes score of 5, which allowed the diagnosis of chronic lymphocytic leukemia-type monoclonal B-cell lymphocytosis (CLL-type MBL) (18).

Fluorescence *in situ* hybridization (FISH) using break-apart probes was performed, revealing a *c-MYC* translocation (without concomitant *BCL2* or *BCL6* rearrangement) in the submandibular gland but not in the SLL/CLL population. No additional translocations, such as t(11;14) or t(14;18), were detected. Thereby cytogenetically confirming the diagnosis of Burkitt lymphoma.

Given the Burkitt lymphoma’s rapid development, a molecular analysis was conducted to investigate the clonal relationship between the Burkitt lymphoma present in the submandibular gland and the SLL/CLL present in the bone marrow. Remarkably, the malignant B-cells found in the submandibular gland and the bone marrow exhibited an identical monoclonal rearrangement of 108bp length in the framework region (FR3 region) of the immunoglobulin heavy chain (*IGH*) locus (**Figures 1L, M**), however an additional peak was noted in the Burkitt lymphoma sample (**Figure 1M**). To further address the question of clonal relatedness and evolution, we performed high-throughput sequencing of the FR3-region of the immunoglobulin heavy (*IGH*) and light chains (*IGK* and *IGL*). *IGH* sequencing partially confirmed the results obtained suing fragment analysis: the SLL/CLL B-cells and the Burkitt lymphoma cells shared the same top rearrangement, with a frequency of 80.1% in the SLL/CLL sample and 40.5% in the Burkitt lymphoma sample (**Figure 1N**; **Table 2**, *IGHV3-11*). Nonetheless, the Burkitt lymphoma cells showed a second dominant rearrangement (39.5%), which was not present in the SLL/CLL cells (**Figures 1N, O**; **Table 2**, *IGHV4-34*). The two rearrangements did not share CDR3 sequence similarity and they did not utilize the same *VJ-*genes (**Table 2**). The result of the immunoglobulin light chains sequencing showed somewhat dissimilar results: the top rearrangement in the CLL/SLL sample (71.8%, *IGKV1-39*), was still present in the Burkitt lymphoma sample with 8.9% (**Table 2**). There was instead another dominant rearrangement in the Burkitt lymphoma sample (79.6%, **Table 2**, *IGKV2-30*), that was not detectable in the SLL/CLL sample. Analysis were in accordance to previously published methods (19–21).

**Table 2:** BCR sequencing: *V* and *J* gene utilization for the top *IGH* and *IGKL* clones for the two tumors, ordered by the occurrence in the Burkitt lymphoma (BL) sample.

	Variable gene	Joining gene	CDR3 amino acids	Frequency in BL	Frequency in SLL/CLL
*IGH*	*IGHV3-11*	*IGHJ4*	AREREGRGWFPSNPADY	40.54%	80.14%
	*IGHV4-34*	*IGHJ4*	STQTWPNSGWDRYADY	39.49%	0%
	*IGHV3-11*	*IGHJ4*	ARERGEGLVPE*PR*L	3.9%	7.8%
	*IGHV3-11*	*IGHJ4*	AREREGEGLVPE*PR*L	2.6%	0%
	*IGHV3-11*	*IGHJ4*	AREREGRAGSRVTPLT	2.08%	7.9%

	Variable gene	Joining gene	CDR3 amino acids	Frequency in BL	Frequency in SLL/CLL
*IGKL*	*IGKV2-30*	*IGKJ2*	MQGTHWPEH	79.6%	0%
	*IGKV1-39*	*IGKJ2*	QQSYSTPPFT	8.9%	71.8%
	*IGKV1-33*	*IGKdel*	none	1.54%	0.009%
	*IGLV7-46*	*IGLJ2*	LLSYNGVRI|LTLL*WCSD	0.79%	0%
	IGKV3-20	*IGKJ1*	QQYGSSRV	0.48%	0%

BCR B-cell receptor, CDR3 complementary determining region, IGH immunoglobulin heavy chain, IGKL immunoglobulin light chain kappa/lambda.

Considering the histopathological, flow cytometric and radiographic findings a Burkitt lymphoma stage I was diagnosed on the background of an indolent CLL-type MBL. This finding suggested an atypical Richter transformation.

Considering the aggressive nature of the transformed lymphoma and the biologically younger patient, an interdisciplinary tumor board recommended an intensive treatment regimen of six cycles DA-EPOCH-R (dose adjusted etoposide prednisone vincristine cyclophosphamide doxorubicin rituximab), starting at a level -1 dose (augmented till dose 2, vincristine 50%). The treatment was well tolerated and the patient responded well clinically.

After administrating six cycles of DA-EPOCH-R treatment, two re-staging PET/CT were conducted, visualizing a complete remission of the previously identified lesions. Regular flow cytometric evaluation of peripheral blood, could not visualize minimal residual disease. As of the twelve-month mark from the initiation of treatment, there are no clinical or laboratory-based signs indicating a relapse.

## Patient perspective

The patient’s ability to articulate the progression of the swelling on her neck and the subsequent diagnostic procedures was remarkable. She expressed significant concern about the possibility of two malignancies and found the idea of transformation from one into the other to be frightening. According to her, engaging in extensive conversations with the medical team proved to be helpful to process the information. While the chemotherapy did result in some fatigue, it had minimal side effects that had a limited impact on her overall quality of life. She experienced a profound sense of relief and gratitude upon receiving a negative PET-CT scan result after completing the last cycle of chemotherapy.

## Discussion

In this case study, the rapid growth of the swelling suggested an aggressive underlying disease, prompting a biopsy. Our analysis revealed a Burkitt lymphoma, localized in the submandibular gland and adjacent lymph node. Given the infiltration of the bone marrow with B-cells with a CLL phenotype and the absence of documented treatment naïve cases of SLL/CLL transforming into a Burkitt lymphoma, clonality was investigated. The analysis, demonstrated a clonal relationship at the molecular level by DNA fragment analysis. A more rigorous approach to investigate the clonal relatedness of both tumors is to sequence the *IGH* and *IGKL* loci. The most dominant rearrangement detected on *IGH*-level in the Burkitt lymphoma sample is the same dominant rearrangement found in the SLL/CLL cells in the bone marrow (*IGHV3-11*). However, the Burkitt lymphoma sample shows a second rearrangement (*IGHV4-34*), that is not detected in the SLL/CLL sample. *IGKL* sequencing results similarly show a shared high-frequency rearrangement between the Burkitt lymphoma and the SLL/CLL sample. However, the frequency of this rearrangement is only 8.9% in the Burkitt lymphoma sample (*IGKV1-39*), which shows a dominant rearrangement at 79.6%, that is absent in the SLL/CLL sample (*IGKV2-30*). These findings are difficult to interpret, and the differential results obtained sequencing immunoglobulin heavy and light chains could be explained considering PCR-amplification biases and somatic hypermutation. The results could indicate the presence of bi-allelic rearrangement, that evolved during the Burkitt lymphoma transformation. That conclusion is supported by the comparable frequency of the *IGHV3-11* and *IGHV4-34* rearrangements (40% *versus* 39.5%). Nevertheless, this could also indicate the presence of a second and distinct clonal population, present only in Burkitt lymphoma sample and present at high frequency according to IGKL sequencing results (*IGKV2-30).* Contamination of reactive B-cells or SLL/CLL cells in the Burkitt lymphoma sample as explanation is less likely, as Burkitt lymphomas are composed up to 90-95% of lymphoma cells (1). The shared clonality renders the hypothesis of the co-incidence of two independent tumors unlikely.

Stereotyped or biased *IGHV* gene usage has been observed previously by high-throughput BCR sequencing in Burkitt lymphoma (22, 23). The clonal expansion of *IGHV3-11* which we observed, was described as stereotype BCR in CLL (24) and in a small proportion of endemic Burkitt lymphoma (23). The second most common *V-*gene segment in the Burkitt lymphoma sample was found to be *IGHV4-34*. *IGHV4-34* expressing clones are commonly found in the naïve B-cell pool but are seldomly present in memory B-cells from healthy persons. The *IGHV4-34* gene segment is intrinsically self-reactive (recognizing I/i carbohydrates expressed on erythrocytes (25, 26), the antibodies additionally cross-react with antigens found on commensal bacterial (27) and the gene segment is one of the most abundant found in CLL patients (28). Contentious observations have been made regarding sporadic BL and the expansion of *IGHV4-34* (22, 28). These findings could imply that certain BCR and thus their antigenic specificities in Burkitt lymphoma precursors are selected and confer a survival advantage. Antigenic stimulation is discussed in the pathogenesis for other malignant B-cell neoplasia and Burkitt lymphoma (22, 23, 28–30). Further patient characterization of BCR Ig stereotypy is needed to investigate antigen-reactivity profiles and similar clinical outcomes stratifications.

In conclusion, this is one of the first *bona fide* descriptions of a treatment naïve patient with CLL-type MBL transforming into a very aggressive Burkitt lymphoma. Further investigations are needed to unveil the molecular drivers and/or genetic predispositions for atypical Richter transformation from indolent SLL/CLL to aggressive Burkitt lymphoma.

## Data availability statement

The raw data supporting the conclusions of this article will be made available by the authors, without undue reservation.

## Ethics statement

The studies involving humans were approved by ethical committee of Northwestern and central Switzerland. The studies were conducted in accordance with the local legislation and institutional requirements. The participants provided their written informed consent to participate in this study. Written informed consent was obtained from the individual(s) for the publication of any potentially identifiable images or data included in this article.

## Author contributions

AJ: Conceptualization, Data curation, Investigation, Supervision, Visualization, Writing – original draft, Writing – review & editing. AR: Resources, Writing – review & editing. AB: Formal analysis, Visualization, Writing – review & editing. IA: Formal analysis, Validation, Writing – review & editing. CR: Formal analysis, Validation, Writing – review & editing. OB: Formal analysis, Writing – review & editing. SD: Formal analysis, Supervision, Validation, Visualization, Writing – review & editing. JP: Supervision, Writing – review & editing. FK: Supervision, Writing – review & editing.
